# Effects of Insect-Proof Net Cultivation, Rice-Duck Farming, and Organic Matter Return on Rice Dry Matter Accumulation and Nitrogen Utilization

**DOI:** 10.3389/fpls.2017.00047

**Published:** 2017-01-24

**Authors:** Xin Liu, Guochun Xu, Qiangsheng Wang, Yuhao Hang

**Affiliations:** Key Laboratory of Crop Physiology, Ecology and Production Management, Ministry of Agriculture, Nanjing Agricultural UniversityNanjing, China

**Keywords:** rice-duck farming, insect-proof net cultivation, organic matter, dry matter accumulation, N utilization

## Abstract

Insect-proof net cultivation (IPN), rice-duck farming (RD), and organic matter return (OM) are important methods to realize sustainable development of rice production. A split-plot field experiment was performed to study the effects of IPN, RD, and OM on the rice yield, dry matter accumulation and N utilization. Results showed that compared to inorganic N fertilizer (IN), wheat straw return, and biogas residue return increased the rice yield by 2.11–4.28 and 4.78–7.67%, respectively, and also improved dry matter and N accumulation after the elongation stage (EG), dry matter and N translocation, and N recovery efficiency (NRE). These results attributed to an increase in leaf SPAD values and net photosynthetic rate (*Pn)* after the EG. Compared to conventional rice farming (CR), RD promoted the rice yield by 1.52–3.74%, and contributed to higher the leaf photosynthesis, dry matter and N accumulation, dry matter and N translocation, and NRE. IPN decreased the intensity of sun radiation in the nets due to the coverage of the insect-proof nets, which declined the leaf *Pn*, dry matter accumulation and translocation, N absorption and translocation, and NRE compared to open field cultivation (OFC). The rice yield of IPN were 2.48–4.98% lower than that of OFC. Compared to the interaction between CR and IN, the interaction between RD and OM improved the rice yield by 5.26–9.33%, and increased dry matter and N accumulation after the EG, dry matter and N translocation, and NRE. These results indicated that OM, RD and the interaction between RD and OM could promote dry matter accumulation and N utilization, which was beneficial to improve the rice yield.

## Introduction

Rice is one of the main food crops worldwide and plays an important role in global food production and consumption. Over the past 60 years, food production has been greatly improved through the use of high-yield varieties and modern fertilizers, irrigation and pesticides (Zeng et al., 2012). However, with the continuous increasing world population, food security has become an increasingly important concern. Improvement in rice production is essential for ensuring global food security (Hu et al., 2013). It is estimated that, to satisfy the rapid growth of population in rice consuming countries by 2030, rice production should be increased by 40% (Khush, 2005). Furthermore, along with the decrease of agricultural land area and continuing of environmental deterioration (Kant et al., 2012), China and other developing countries are facing the dual challenge of increasing rice yield while at the same time reducing environmental threats (Chen et al., 2011). Rice yield is comprehensively influenced by cultivation environment, soil nutrients, and field management. Field management is easily controlled by human factors. Thus, the improvement of field management plays an important role in increasing rice yield.

Rice diseases and insect pests are the major limiting barriers of yield. Chemical pesticides and fungicides are commonly used to prevent diseases and insect pests to avoid yield loss. However, pesticides and fungicides can be retained in the surface water or soil, which may diminish the effectiveness and cause serious harm to the environment. Insect-proof nets provide an ecological and effective approach for controlling the infection and transfer of plant diseases and insect pests (Guo et al., 2015). RD is a mode in which a certain number of ducks are raised in a rice field to eat weeds, insects, and small aquatic animals (Xu et al., 2017). Additionally, the ducks wander while feeding and excreting, which is helpful for intertilling, weeding, building soil fertility, and stimulating rice growth (Suh, 2014). RD, which is highly praised by rice growers, rice consumers, and government, has been known as the best ecological method for developing sustainable agriculture.

Rice-wheat rotation is the dominant farming system in the Yangtze River region of China, which can produce large amounts of straw residue (Wang X.H. et al., 2015). However, due to the transfer of rural labor, some farmers directly burn straw to save time. But burning causes severe environment pollution and soil degradation and thus it is forbidden by law in China (Zhang et al., 2014). Returning straw into the soil may be an effective agricultural practice (Seufert et al., 2012). This method not only solves environmental problems but also promotes the nutrient recycling and sustainable environmental development. Previous studies have indicated that straw return was an effective means to improve soil quality and rice yield (Liu et al., 2014). BR is the solid residue that remains after the anaerobic fermentation of organic wastes, including crop straw and human and animal excreta, and contains N, P, K, calcium, magnesium, humic acid, organic acid, and cellulose (Liu et al., 2010). Thus, BR is a high-quality organic fertilizer.

Currently, through a series of subsidy policies, the government advocates wheat straw and organic fertilizer return. In Jiangsu, China, more than 70% of the total rice-wheat growing region practices straw return. As the key technology in rice ecological control, insect-proof net mulching has attracted significant attention and has been applied by agricultural workers. However, few studies on the effects of insect-proof net mulching, RD, and OM on rice yield and population quality have been conducted. The objectives of this study were to investigate the effects of IPN, RD, and OM on rice dry matter accumulation and N utilization, and to further explore the relationships between dry matter accumulation, N utilization and rice yield in rice production.

## Materials and Methods

### Site Descriptions

The field experiments were conducted at the Baiwei Farm of Nanjing Agricultural University (32°34′ N, 120°24′ E) from 2014 to 2015. The experimental region is characterized by a subtropical monsoon climate. The annual mean temperature at Baiwei farm is 14.5°C; the mean temperature during the rice growing season is 22.5°C; the annual mean precipitation is 1025 mm; the annual total solar radiation is 4.99 × 10^9^ J m^-2^; and the annual total solar radiation during the rice growing season is 3.01 × 10^9^ J m^-2^. The fore-rotating crop was wheat, and the soil was clay, with soil properties as follows: organic matter 24.6 g kg^-1^, total N 1.26 g kg^-1^, available N 97.2 mg kg^-1^, available P 24.3 mg kg^-1^, and available K 95.7 mg kg^-1^.

### Experimental Design

Using a split-plot design, the experiments took cultivation environment as main plot, and OFC and IPN as two treatments. IPN used a rigid frame and a flat roof covered with white nets on the outside for insect proofing. Using cultural practice as subplot, the experimental design included two treatments, i.e., CR and RD. Fertilizer management was used as sub-subplot, including IN and OM, and OM refers to WS and BR.

The experiment was performed with equal amounts of nutrients. The amount of WS to the soil was 6000 kg ha^-1^, and the amount of BR after fermentation of wheat straw was 10,500 kg ha^-1^. Both wheat straw and biogas residue were used as base fertilizers. All treatments received the same amount of nutrient in rice season, including 300 kg N ha^-1^, 150 kg P_2_O_5_ ha^-1^, and 150 kg K_2_O ha^-1^, and deficient nutrients were supplemented using inorganic fertilizer. N was applied as follows: 15% as base fertilizer, 45% as tiller fertilizer, and 40% as panicle fertilizer. Tiller fertilizer was used in an equal amount and applied on the 7th day and 14th day after transplanting. P_2_O_5_ was used entirely as base fertilizer, and K_2_O was used as base fertilizer and panicle fertilizer at equal amounts. To calculate the N utilization efficiency in each treatment, an additional treatment was established in which N was not applied but P_2_O_5_ and K_2_O were added.

The experimental variety was Nanjing9108, which was sown on May 24th, and seedlings by substrate nursing were mechanically transplanted on June 15th with a hill spacing of 13.3 cm × 30 cm and four seedlings per hole. The experiment was performed in three replicates with the plot area of 200 m^2^ (16 m × 12.5 m); the plots were separated by ridges using plastic film, and the irrigation and drainage in each plot were performed separately. Ducklings were introduced into the RD area with a density of 225 ducks ha^-1^ on the 17th day after transplanting. The RD fields were surrounded by nylon nets (1 m in height) to prevent the ducks from escaping, and a shed for the ducks was also built in the corner of each RD plot. The ducks were retrieved at the HD. A standing water of about 5–8 cm was maintained in the field during the period of raising ducks.

### Parameter Measurements

#### Climatic Conditions

The wind speed and CO_2_ concentration during the rice growing period from May to October were provided by the local Meteorological Station. From the booting stage to the grain filling stage, three weather types were chosen, i.e., sunny days, cloudy days, and overcast days to measure light intensity by an illuminometer (TES1339, Lexian Electronic Technology Company, China). For 3 days, the light intensity was tested simultaneously on each day at 20 cm above the rice canopy inside and outside the nets in the morning (9:00–10:00), at noon (12:00–13:00), and in the afternoon (15:00–16:00), and the light intensity was tested five times at 10-min intervals.

#### Chlorophyll Content

A SPAD-502 chlorophyll meter was used to estimate the SPAD values of the top leaf (all of the expanding leaves on the top) at the main growth stages, i.e., the TP, ETC, EG, HD and 30 days after transplanting (30 DAH). Thirty leaves in each treatment were chosen to determine the chlorophyll contents at the upper, middle and lower positions, and the mean values were used.

#### Net Photosynthetic Rate and Transpiration Rate

On sunny days between 10:00 and 11:00, 10 plants in each treatment were chosen to determine the net *Pn, Tr, Gs*, and *Ci* in the top leaf (all of the expanding leaves on the top) by a gas exchange analyser (Li-6400, Li-COR, Inc., Lincolin, NE, USA) at the main growth stages, i.e., the ETC, EG, HD, and 30 DAH. For environmental factors with a relatively large influence on gas exchange parameters, before determining the leaf gas exchange parameter, the environmental conditions were controlled as follows: the flow rate was 500 μmol s^-1^, the CO_2_ concentration was 380 μmol mol^-1^, the temperature of leaf chamber was within ± 6°C of atmosphere temperature, and the photosynthetic active radiation intensity was 1200 μmol m^-2^ s^-1^.

### Dry Matter Accumulation and N Content

Five holes of representative plants were chosen in each plot at the ETC, EG, HD, and MT. After the stems, leaves, and panicles (HD and MT) were separated, fresh samples were killed out at 105°C for 30 min and then oven-dried at 80°C until a constant weight was reached to determine the dry matter weight. Then, the samples were milled and sieved to determine their total N content by using the Kjeldahl method.

### Yield Determination

In each plot, 65 m^2^ of rice was chosen to determine the actual yield and yield components, which mainly refer to the effective panicle number, number of grains per panicle, seed-setting rate and grain weight at the mature stage.

### Analysis Methods

The dry matter or N accumulation rate (kg⋅ha^-1^⋅d^-1^) = the D-value of dry matter or N accumulation in the two aboveground samples/the interval time between the two samples.

The amounts of apparent dry matter or N translocation from vegetative organs after the heading stage (DT or NT, respectively, kg⋅ha^-1^) = the amounts of dry matter or N accumulation in the aboveground vegetation at the heading stage – the amounts of dry matter or N accumulation in the aboveground vegetation at the mature stage.

The apparent dry matter or N translocation efficiency from vegetative organs after the heading stage (DTE or NTE, respectively, %) = the amounts of apparent dry matter or N translocation from vegetative organs after the heading stage/the amounts of dry matter or N accumulation in the aboveground vegetation at the heading stage.

The contribution rates of the transferred dry matter or N from the vegetative organs to grain after the heading stage (DCR or NCR, respectively, %) = the amounts of apparent dry matter or N translocation from the vegetative organs after the heading stage/the amounts of dry matter or N accumulation in the grains at the mature stage.

The N recovery efficiency (NRE, %) = (the total N uptake in the N application area – the total N uptake in the area without N application)/the amount of N application × 100.

The N agronomic efficiency (NAE, %) = (the rice yield in the N application area – the rice yield in the area without N application)/the amount of applied N × 100.

The N physiological efficiency (NPE, kg⋅kg^-1^) = (the rice yield in the N application area – the rice yield in the area without N application)/(the total N uptake in the N application area – the total N uptake in the area without N application).

The N grain production efficiency (NGPE, kg⋅kg^-1^) = the rice yield/the total N uptake.

The N dry matter production efficiency (NDMPE, kg⋅kg^-1^) = the accumulation of aboveground dry matter at the mature stage/the total N uptake.

The N uptake per 100 kg of grains (NUG, 100 kg kg^-1^) = the total N uptake/the rice yield.

### Data Analysis

SPSS and Office 2007 were used to process and analyze the data, and the results were expressed as the mean values of three replicates. Least significant difference (LSD) tests were used to compare the means for each treatment in the same year. Origin 8.1 was used to visualize the data, and the standard errors of the means were calculated and presented in the graphs as error bars. Analyses of variance (*F*-value) of rice leaf photosynthesis, dry matter accumulation, N absorption, and N utilization efficiency were performed. Then, linear relationships between the rice yield and the dry matter accumulation, N absorption and N utilization efficiency, and the significance probability levels of the results were given at ^∗^*P* < 0.05 and ^∗∗^*P* < 0.01, respectively. Based on the data analysis summarized in **Table 1**, the results for 2014 and 2015 showed a similar trend; accordingly, except for the rice yield and yield components, the subsequent analyses described in the text focused on the 2014 data.

**Table 1:** Analysis of variance of yield with different years and treatments.

Source of variation	*Df*	Yield
Year	1	ns
Treatments	11	^∗∗^
Year × Treatments	11	ns

*^∗∗^*P* < 0.01, ^∗^*P* < 0.05, ns, non-significant at *P* > 0.05.*

### Ethics Statement

This study was carried out in accordance with the Guidelines for Experimental Animals established by Ministry of science and technology of the People’s Republic of China. All experimental protocols were approved by Animal Ethics committee of Nanjing Agricultural University (Nanjing, China).

## Results

### Wind Speed, CO_2_ Concentration, and Light Intensity

**FIGURE 1:**
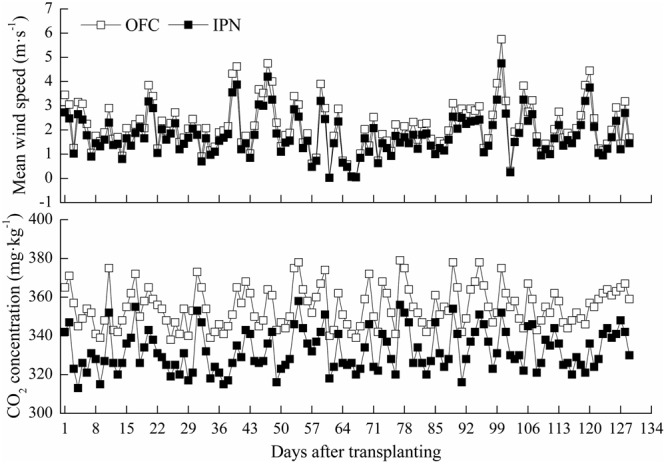
**The effects of IPN on the wind speed and CO_2_ concentration of the rice fields.** OFC, open field cultivation; IPN, insect-proof net cultivation.

Insect-proof net cultivation had significant effect on wind speed, light intensity and CO_2_ concentration. The wind speed of IPN was 0.01–0.73 m s^-1^ lower than that of OFC, and CO_2_ concentration of IPN was decreased by 3.62–9.52% compared to OFC (**Figure 1**). Regardless of what the weather type was, IPN significantly decreased the light intensity in the nets compared to OFC (**Table 2**).

**Table 2:** The effects of IPN on the light intensity of the rice canopy.

Measurement time		Light intensity (Lx)		Light intensity decline (%)
		OFC	IPN	
Sunny days	Morning (9:00–10:00)	84870.22	62181.52	26.73
	Noon (12:00–13:00)	119443.71	82536.66	30.90
	Afternoon (15:00–16:00)	74904.68	55920.38	25.34
Cloudy days	Morning (9:00–10:00)	37859.64	28673.06	24.26
	Noon (12:00–13:00)	44567.80	31947.21	28.32
	Afternoon (15:00–16:00)	26799.44	19748.78	26.31
Overcast days	Morning (9:00–10:00)	8968.17	6948.31	22.52
	Noon (12:00–13:00)	11014.66	8395.60	23.78
	Afternoon (15:00–16:00)	9323.21	7489.68	19.67

*OFC, open field cultivation; IPN, insect-proof net cultivation.*

### Rice Yield

**FIGURE 2:**
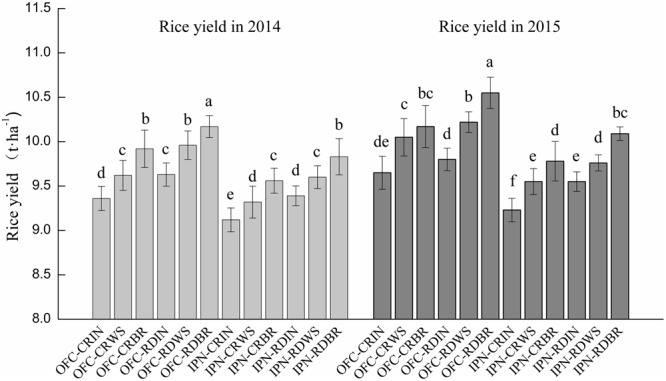
**The effects of IPN, RD, and OM on the rice yield.** IN, inorganic N fertilizer; WS, wheat straw return; BR, biogas residue return; CR, conventional rice farming; RD, rice-duck farming; OFC, open field cultivation; IPN, insect-proof net cultivation. Different letters above the column indicate significant differences at *P* < 0.05. Vertical bars represent the standard errors of means.

There were significant differences in rice yields between IN and OM (**Figure 2**). The rice yields of WS and BR were 2.11–4.28 and 4.78–7.67% higher than that of IN, respectively. The higher rice yield of WS was mainly attributed to more grains per panicle, and the greater rice yield of BR was attributed to the more effective panicle number or grain number per panicle (**Table 3**). The rice yields showed significant differences between CR and RD. The rice yield of RD was 1.52–3.74% higher than that of CR, mainly because of the more effective panicle number, grain number per panicle, seed-setting rate and grain weight.

**Table 3:** The effects of IPN, RD, and OM on the yield components of rice.

Treatments	Yield components of rice in 2014				Yield components of rice in 2015			
	Effective panicle number (× 10^4^ ha^-1^)	No. of grains per panicle	Seed-setting rate (%)	1000-grain weight (g)	Effective panicle number (× 10^4^ ha^-1^)	No. of grains per panicle	Seed setting rate (%)	1000-grain weight (g)
OFC-CRIN	338.55abc	134.1d	93.27c	26.11c	344.10bcd	132.9d	95.27bc	26.32e
OFC-CRWS	330.23c	140.7a	92.63e	26.20c	335.78d	138.4a	94.63d	26.50d
OFC-CRBR	344.10ab	135.2bc	93.11cd	26.55b	352.43ab	134.2bc	95.21bc	26.63c
OFC-RDIN	344.10ab	134.3cd	93.51b	26.48b	346.88abc	133.2cd	95.41ab	26.53cd
OFC-RDWS	335.78bc	141.1a	93.03d	26.53b	341.33cd	138.7a	95.03c	26.82b
OFC-RDBR	346.88a	135.7b	93.77a	26.71a	355.20a	134.6b	95.77a	26.93a
***F*-value**								
Mean	339.94	136.85	93.22	26.43	345.95	135.33	95.22	26.62
CP	175.18^∗∗^	5.95	213.92^∗∗^	150.94^∗∗^	4.41	78.74^∗^	25.80^∗^	166.67^∗∗^
FM	6.20^∗^	267.99^∗∗^	58.16^∗∗^	42.99^∗∗^	26.04^∗∗^	83.60^∗∗^	33.82^∗∗^	110.75^∗∗^
CP × FM	0.10	0.12	5.70^∗^	4.28	0.29	0.01	3.17	2.92

IPN-CRIN	321.90ab	133.3c	92.45d	26.04d	330.23c	131.1c	94.55c	26.23d
IPN-CRWS	316.35b	139.2ab	92.10e	26.12cd	321.90d	136.4ab	94.10d	26.48c
IPN-CRBR	330.23ab	135.7bc	92.82b	26.26bc	338.55ab	133.5bc	94.72c	26.62b
IPN-RDIN	327.45ab	133.5c	92.91b	26.42ab	333.00bc	131.6c	95.01b	26.41c
IPN-RDWS	319.13ab	140.3a	92.63c	26.30bc	327.45cd	137.9a	94.63c	26.64b
IPN-RDBR	335.78a	136.1abc	93.22a	26.51a	341.33a	133.8bc	95.42a	26.82a
***F*-value**								
Mean	325.14	136.35	92.69	26.28	332.08	134.05	94.74	26.53
CP	1.52	0.41	320.24^∗∗^	40.25^∗^	11.26	1.37	294.44^∗∗^	168.23^∗∗^
FM	12.59^∗∗^	20.97^∗∗^	147.45^∗∗^	14.39^∗∗^	42.04^∗∗^	95.22^∗∗^	101.01^∗∗^	91.20^∗∗^
CP × FM	0.14	0.11	1.45	4.04	0.46	1.15	3.06	0.22

*IN, inorganic N fertilizer; WS, wheat straw return; BR, biogas residue return; CR, conventional rice farming; RD, rice-duck farming; OFC, open field cultivation; IPN, insect-proof net cultivation; CP, cultural practice (conventional rice farming and rice-duck farming); FM, fertilizer management (inorganic N fertilizer, wheat straw return and biogas residue return). Values within a column followed by different letters are significantly different at *P* < 0.05. ^∗∗^*P* < 0.01 and ^∗^*P* < 0.05.*

Compared to OFC, IPN significantly decreased the rice yield by 2.48–4.98% due to a lower effective panicle number (**Figure 2**; **Table 3**), which suggested that insect-proof net mulching was not beneficial for rice yield. The rice yield of the interaction between RD and OM was increased by 5.26–9.33% compared to the interaction between CR and IN due to the higher grain number per panicle of WS and the greater effective panicle number, grain number per panicle and grain weight of BR (**Table 3**). The rice yields of 2014 were 1.19–4.49% lower than those of 2015. The lower rice yields in 2015 mainly resulted from the temperature during the later stages of growth in 2014, which was not beneficial for rice growth.

### Photosynthesis in Leaves

**FIGURE 3:**
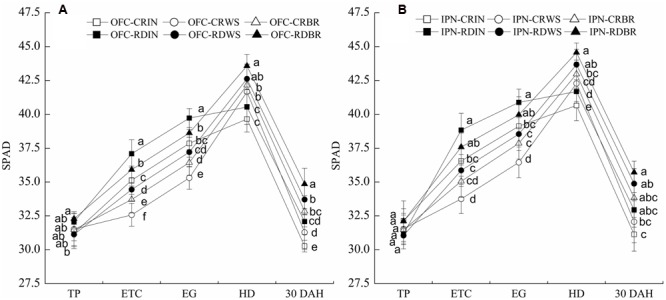
**The effects of IPN, RD, and OM on the leaf SPAD values of rice.** (**A**: open field cultivation) and (**B**: insect-proof net cultivation). TP, transplanting stage; ETC, effective tiller critical leaf stage; EG, elongation stage; HD, heading stage; 30 DAH, 30 days after heading; IN, inorganic N fertilizer; WS, wheat straw return; BR, biogas residue return; CR, conventional rice farming; RD, rice-duck farming; OFC, open field cultivation; IPN, insect-proof net cultivation. Different letters in the figure indicate significant differences at *P* < 0.05. The vertical bars represent the standard errors of means.

During rice growth, the SPAD values increased gradually from the TP to HD and peaked at the HD before decreasing (**Figure 3**). No significant difference was found between IN and OM at the TP. The leaf SPAD values of WS and BR were lower than those of IN at the ETC and EG. However, at the HD and 30 DAH, WS increased the leaf SPAD values by 4.01–5.13 and 2.99–5.86%, respectively, and BR increased them by 5.71–7.47 and 8.38–8.64%, respectively, compared to IN. The leaf SPAD values of RD were higher than those of CR. There were significant differences in the leaf SPAD values between CR and RD at the ETC and EG, and RD increased those by 5.54–7.19 and 4.50–5.98%, respectively. Compared to OFC, the leaf SPAD values were increased by IPN, indicating IPN could promote the leaf chlorophyll content. At the ETC and EG, the interaction between RD and WS decreased the leaf SPAD values while the interaction between RD and BR showed the opposite trend. However, at the HD and 30 DAH, the interaction between RD and OM increased the leaf SPAD values compared to the interaction between CR an IN.

In the rice growth process, the leaf *Pn, Tr*, and *Gs* initially increased, peaked at the HD and then decreased, while *Ci* exhibited the opposite pattern (**Table 4**). In contrast with IN, the *Pn, Tr, Gs* of WS and BR were lower at the ETC and EG. However, at the HD and 30 DAH, both WS and BR contributed to higher leaf *Pn, Tr*, and *Gs* than those of IN, while the trend of *Ci* was the opposite. At the ETC, EG, HD and 30 DAH, the *Pn, Tr*, and *Gs* of RD were higher than those of CR, and *Ci* exhibited the opposite pattern. For the coverage with insect-proof nets, the leaf *Pn, Tr*, and *Gs* of IPN were decreased but the *Ci* was increased compared to OFC. Regarding the interaction between RD and OM, there was no significant influence on leaf photosynthetic characteristics. The *Pn, Tr*, and *Gs* of the interaction between RD and OM were all higher than those of the interaction between CR and IN, while *Ci* showed the opposite trend.

**Table 4:** The effects of IPN, RD, and OM on the leaf *Pn, Tr, Gs*, and *Ci* of rice.

Treatments	*Pn* (μmol.m^-2^.s^-1^)				*Tr* (μmol.m^-2^.s^-1^)					*Gs* (μmol.m^-2^.s^-1^)				*Ci* (μmol.mol^-1^)			
	ETC	EG	HD	30 DAH	ETC	EG	HD	30 DAH	ETC	EG	HD	30 DAH	ETC	EG	HD	30 DAH
OFC-CRIN	14.26b	16.19c	18.55d	12.31d	7.85cd	9.63c	11.53e	6.36d	0.41ab	0.45c	0.47c	0.32c	280.25b	273.32bc	271.53a	300.57a
OFC-CRWS	12.68d	15.43d	19.25cd	12.55d	6.26e	8.74d	12.26d	8.28c	0.38b	0.42d	0.49c	0.35bc	292.38a	284.22a	268.39ab	293.05ab
OFC-CRBR	13.35c	16.02c	19.48c	13.22c	7.41d	9.17cd	12.93c	8.74bc	0.39ab	0.44cd	0.53b	0.41ab	285.47b	276.56ab	265.27abc	291.37bc
OFC-RDIN	16.31a	18.74a	20.59b	14.25b	9.64a	11.91a	13.16c	8.95bc	0.49a	0.52a	0.54b	0.41ab	269.49c	262.38d	264.68abc	285.43bcd
OFC-RDWS	14.81b	17.62b	21.37a	14.56b	8.21c	10.84b	14.55b	9.27b	0.44ab	0.49b	0.58a	0.43a	273.32c	268.29bcd	261.35bc	283.47cd
OFC-RDBR	15.73a	18.53a	21.64a	15.54a	8.93b	11.62a	15.37a	10.21a	0.48ab	0.50ab	0.61a	0.45a	270.25c	265.21cd	259.74c	280.54d
***F*-value**																
Mean	14.52	17.09	20.15	13.74	8.05	10.32	13.30	8.64	0.43	0.47	0.54	0.40	278.53	271.66	265.16	289.07
CP	340.10^∗∗^	361.42^∗∗^	168.46^∗∗^	274.85^∗∗^	232.63^∗∗^	262.51^∗∗^	253.39^∗∗^	125.50^∗∗^	10.80	300.00^∗∗^	144.00^∗∗^	27.00^∗^	172.19^∗∗^	67.38^∗^	86.94^∗^	53.45^∗^	
FM	52.36^∗∗^	172.02^∗∗^	35.63^∗∗^	48.42^∗∗^	43.53^∗∗^	58.80^∗∗^	113.84^∗∗^	41.90^∗∗^	5.35^∗^	7.36^∗^	24.58^∗∗^	19.35^∗∗^	19.33^∗∗^	4.52^∗^	2.75	9.78^∗∗^
CP × FM	0.65	6.78^∗^	0.13	1.51	0.88	1.84	6.42^∗^	8.38^∗^	0.76	0.27	0.58	3.15	5.13^∗^	0.48	0.06	1.61

IPN-CRIN	13.52d	15.92bc	17.52d	12.02d	7.24cd	9.02cd	10.74e	6.15d	0.35b	0.36b	0.37d	0.29c	291.55ab	281.36a	276.83a	304.26a
IPN-CRWS	12.24e	15.14d	18.29c	12.15cd	5.35e	7.68e	11.67d	7.36c	0.31b	0.33b	0.40cd	0.30c	295.74a	287.52a	274.32ab	302.18a
IPN-CRBR	13.05d	15.55cd	18.71c	12.71c	6.67d	8.45d	12.22c	8.55b	0.33b	0.34b	0.44c	0.32c	293.41ab	285.93a	273.01ab	299.46a
IPN-RDIN	15.83a	17.62a	19.45b	13.82b	9.16a	11.15a	12.95b	8.62b	0.46a	0.47a	0.51b	0.38b	275.34b	268.55b	271.44abc	295.64a
IPN-RDWS	14.29c	16.37b	20.06a	14.13b	7.74bc	9.56c	13.74a	9.02ab	0.42a	0.44a	0.55ab	0.40ab	283.26ab	277.82ab	268.52bc	293.37ab
IPN-RDBR	15.27b	17.11a	20.49a	15.01a	8.28b	10.27b	14.08a	9.73a	0.43a	0.46a	0.58a	0.42a	281.56ab	270.48b	265.43c	282.69b
***F*-value**																
Mean	14.03	16.29	19.09	13.31	7.41	9.36	12.57	8.24	0.38	0.40	0.48	0.35	286.81	278.61	271.59	296.27
CP	391.70^∗∗^	176.69^∗∗^	331.34^∗∗^	283.41^∗∗^	284.16^∗∗^	191.24^∗∗^	324.62^∗∗^	130.30^∗∗^	146.29^∗∗^	385.33^∗∗^	113.20^∗∗^	280.33^∗∗^	10.00	73.83^∗^	25.99^∗^	26.08^∗^
FM	163.85^∗∗^	80.54^∗∗^	44.56^∗∗^	23.22^∗∗^	42.33^∗∗^	48.93^∗∗^	132.97^∗∗^	54.68^∗∗^	3.87	4.76^∗^	9.59^∗∗^	6.00^∗^	4.87^∗^	2.81	4.37	4.82^∗^
CP × FM	1.40	4.53^∗^	0.28	1.54	2.37	0.62	2.34	7.53^∗^	0.08	0.18	0.07	0.16	0.17	0.39	0.24	1.22

*ETC, effective tiller critical leaf stage; EG, elongation stage; HD, heading stage; 30 DAH, 30 days after heading; *Pn*, net photosynthetic rate; *Tr*, transpiration rate; *Gs*, stomatal conductance; *Ci*, intercellular CO_*2*_ concentration; IN, inorganic N fertilizer; WS, wheat straw return; BR, biogas residue return; CR, conventional rice farming; RD, rice-duck farming; OFC, open field cultivation; IPN, insect-proof net cultivation. CP, cultural practice (conventional rice farming and rice-duck farming); FM, fertilizer management (inorganic N fertilizer, wheat straw return and biogas residue return). Values within a column followed by different letters are significantly different at *P* < 0.05. ^∗∗^*P* < 0.01 and ^∗^*P* < 0.05.*

### Dry Matter Accumulation and Translocation

During rice growth, the amount and ratio of dry matter accumulation increased, and the dry matter accumulation rate gradually increased and reached its peak from the EG to HD, then decreased (**Table 5**). Compared to IN, WS, and BR significantly increased the dry matter accumulation at the MT by 7.05–7.50 and 12.17–14.79%, respectively. The differences in the DT, DTE, and DCR were significant between IN and OM. WS and BR increased the DT by 12.72–15.32 and 23.90–27.46%, respectively, and improved the DTE and DCR. WS and BR had lower dry matter accumulation, accumulation ratio and rate than IN from the TP to EG but higher values than IN from the EG to MT.

**Table 5:** The effects of IPN, RD, and OM on the dry matter accumulation and translocation characteristics of rice.

Treatments	Dry matter accumulation (t.ha^-1^)					Dry matter accumulation ratio (%)				Dry matter accumulation rate (kg.ha^-1^.d^-1^)				Dry matter translocation from vegetative organs after heading		
	TP–ETC	ETC–EG	EG–HD	HD–MT	MT	TP–ETC	ETC–EG	EG–HD	HD–MT	TP–ETC	ETC–EG	EG–HD	HD–MT	DT (kg.ha^-1^)	DTE (%)	DCR (%)
OFC-CRIN	1.48abc	2.43abc	5.77d	6.67e	16.35e	9.05a	14.86a	35.30a	40.79b	42.29b	162.00abc	213.70d	133.4f	2178.99e	22.55d	24.63e
OFC-CRWS	1.36c	2.36c	6.59c	7.21d	17.52d	7.76c	13.47bc	37.62a	41.15b	38.86d	157.33c	244.07c	144.2e	2501.57d	24.29c	25.74cd
OFC-CRBR	1.41bc	2.42bc	6.93bc	7.58c	18.34c	7.69c	13.19bc	37.80a	41.32ab	40.29c	161.33bc	256.67bc	151.6d	2699.72c	25.12c	26.29bc
OFC-RDIN	1.54a	2.58a	6.52c	7.74c	18.38c	8.38b	14.03ab	35.48a	42.11ab	44.00a	172.00a	241.48c	154.8c	2667.67c	25.11c	25.59d
OFC-RDWS	1.49ab	2.47abc	7.41ab	8.38b	19.75b	7.54c	12.50cd	37.53a	42.43ab	42.57b	164.67abc	274.44ab	167.6b	3037.02b	26.73b	26.59b
OFC-RDBR	1.53ab	2.52ab	7.92a	9.11a	21.08a	7.25c	11.96d	37.56a	43.23a	43.71a	168.00ab	293.33a	182.2a	3392.51a	28.35a	27.20a
***F*-value**																
Mean	1.47	2.46	6.86	7.78	18.57	7.95	13.34	36.88	41.84	41.95	164.22	253.95	155.63	2746.25	25.36	26.01
CP	29.72^∗^	105.08^∗∗^	113.58^∗∗^	284.07^∗∗^	283.40^∗∗^	71.22^∗^	45.44^∗^	0.04	26.55^∗^	29.72^∗^	105.08^∗∗^	113.58^∗∗^	284.07^∗∗^	271.87^∗∗^	203.62^∗∗^	66.91^∗^
FM	2.96	1.53	21.82^∗∗^	106.47^∗∗^	252.19^∗∗^	118.19^∗∗^	20.62^∗∗^	4.85^∗^	1.09	2.96	1.53	21.82^∗∗^	106.47^∗∗^	246.09^∗∗^	37.53^∗∗^	121.41^∗∗^
CP × FM	0.59	0.13	0.20	4.79^∗^	6.13^∗^	3.40	0.23	0.03	0.20	0.59	0.13	0.20	4.79^∗^	7.23^∗^	0.79	0.32

IPN-CRIN	1.44ab	2.41ab	5.28d	6.08e	15.21e	9.47a	15.82a	34.73b	39.98c	41.14ab	160.67ab	195.56d	116.92e	1906.87e	20.90e	23.92d
IPN-CRWS	1.32b	2.29b	6.13c	6.61d	16.35d	8.07bc	14.00b	37.51a	40.42bc	37.71b	152.67b	227.04c	127.12d	2199.02d	22.59d	24.95c
IPN-CRBR	1.37b	2.36ab	6.56b	7.17c	17.46c	7.85bc	13.52bc	37.59a	41.04abc	39.14b	157.33ab	242.96b	137.88c	2430.59c	23.63cd	25.46c
IPN-RDIN	1.51a	2.51a	6.13c	7.16c	17.31c	8.72ab	14.50b	35.43b	41.36abc	43.14a	167.33a	227.04c	137.69c	2431.99c	23.98c	25.44c
IPN-RDWS	1.41ab	2.37ab	6.94b	7.81b	18.53b	7.61c	12.79c	37.46a	42.14ab	40.29ab	158.00ab	257.04b	150.19b	2741.37b	25.58b	26.04b
IPN-RDBR	1.43ab	2.46ab	7.38a	8.39a	19.66a	7.27c	12.51c	37.55a	42.67a	40.86ab	164.00ab	273.33a	161.35a	3042.78a	27.01a	26.64a
***F*-value**																
Mean	1.41	2.40	6.40	7.20	17.42	8.17	13.86	36.72	41.27	40.38	160.00	237.16	138.53	2458.77	23.95	25.41
CP	9.68	28.00^∗^	115.61^∗∗^	290.74^∗∗^	276.19^∗∗^	8.85	100.31^∗∗^	0.41	218.28^∗∗^	9.88	28.00^∗^	115.61^∗∗^	290.74^∗∗^	216.45^∗∗^	199.95^∗∗^	147.90^∗∗^
FM	5.05^∗^	2.18	71.37^∗∗^	56.53^∗∗^	192.91^∗∗^	26.23^∗∗^	25.05^∗∗^	15.29^∗∗^	1.71	5.05^∗^	2.18	71.37^∗∗^	56.53^∗∗^	204.59^∗∗^	60.27^∗∗^	66.42^∗∗^
CP × FM	0.09	0.02	0.02	0.24	0.10	0.20	0.13	0.35	0.04	0.09	0.02	0.02	0.24	1.35	0.30	1.89

*TP, transplanting stage; ETC, effective tiller critical leaf stage; EG, elongation stage; HD, heading stage; MT, mature stage; DT, the amount of apparent dry matter translocation from vegetative organs after the heading stage; DTE, apparent dry matter translocation efficiency from vegetative organs after the heading stage; DCR, the contribution rate of the transferred dry matter from vegetative organs to grain after the heading stage; IN, inorganic N fertilizer; WS, wheat straw return; BR, biogas residue return; CR, conventional rice farming; RD, rice-duck farming; OFC, open field cultivation; IPN, insect-proof net cultivation; CP, cultural practice (conventional rice farming and rice-duck farming); FM, fertilizer management (inorganic N fertilizer, wheat straw return and biogas residue return). Values within a column followed by different letters are significantly different at *P* < 0.05. ^∗∗^*P* < 0.01 and ^∗^*P* < 0.05.*

There were significant differences in the DT, DTE, DCR and the dry matter accumulation at the MT (**Table 5**). Compared to CR, RD increased the DT and the dry matter accumulation at the MT by 21.40–27.54 and 12.42–14.94%, respectively. RD had higher DTE, DCR, dry matter accumulation and accumulation rate from the TP to MT, while the dry matter accumulation ratio was lower than that of CR from the TP to EG but higher than that of CR from the EG to MT. However, IPN decreased the DT and the dry matter accumulation at the MT by 8.84–12.49 and 4.80–6.97%, respectively, and it also declined the DTE, DCR and dry matter accumulation compared to OFC. The interaction between RD and OM had no significant influence on the dry matter accumulation and translocation. However, compared to the interaction between CR and IN, the interaction between RD and OM increased the DT and the dry matter accumulation at the MT by 39.38–59.57 and 20.80–29.26%, respectively, and improved the DTE and DCR.

The correlation analysis indicated that the dry matter accumulations from the TP to EG, from the HD to MT and during the MT were positively correlated with the rice yields under IN, WS and BR, respectively (**Table 6**). There were significantly positive correlations between the dry matter accumulation from the EG to MT, during the MT and the rice yields under CR, RD, OFC, and IPN, respectively. The positive correlations between the DT, the DTE, the dry matter accumulation and the rice yield were found. These results suggested that the high dry matter accumulation and translocation were beneficial to enhance the rice yield.

**Table 6:** The effects of IPN, RD, and OM on the correlations between rice yield and the dry matter accumulation and translocation.

Treatments	Dry matter accumulation					Dry matter translocation from vegetative organs after heading		
	TP–ETC	ETC–EG	EG–HD	HD–MT	MT	DT	DTE	DCR
IN (*n* = 12)	0.739^∗∗^	0.652^∗^	0.535	0.902^∗∗^	0.863^∗∗^	0.908^∗∗^	0.857^∗∗^	0.541
WS (*n* = 12)	0.763^∗∗^	0.800^∗∗^	0.440	0.873^∗∗^	0.844^∗∗^	0.855^∗∗^	0.882^∗∗^	0.441
BR (*n* = 12)	0.679^∗^	0.661^∗^	0.540	0.789^∗∗^	0.802^∗∗^	0.772^∗∗^	0.783^∗∗^	0.154
CR (*n* = 18)	0.256	0.361	0.559^∗∗^	0.912^∗∗^	0.903^∗∗^	0.931^∗∗^	0.868^∗∗^	0.542^∗^
RD (*n* = 18)	0.267	0.274	0.684^∗∗^	0.921^∗∗^	0.928^∗∗^	0.908^∗∗^	0.929^∗∗^	0.312
OFC (*n* = 18)	0.388	0.397	0.702^∗∗^	0.880^∗∗^	0.874^∗∗^	0.899^∗∗^	0.884^∗∗^	0.630^∗∗^
IPN (*n* = 18)	0.249	0.380	0.654^∗∗^	0.897^∗∗^	0.891^∗∗^	0. 870^∗∗^	0.882^∗∗^	0.369
AT (*n* = 36)	0.390^∗^	0.434^∗∗^	0.709^∗∗^	0.893^∗∗^	0.893^∗∗^	0.896^∗∗^	0.886^∗∗^	0.511^∗∗^

*TP, transplanting stage; ETC, effective tiller critical leaf stage; EG, elongation stage; HD, heading stage; MT, mature stage; DT, the amount of apparent dry matter translocation from vegetative organs after the heading stage; DTE, apparent dry matter translocation efficiency from vegetative organs after the heading stage; DCR, the contribution rate of the transferred dry matter from vegetative organs to grain after the heading stage; IN, inorganic N fertilizer; WS, wheat straw return; BR, biogas residue return; CR, conventional rice farming; RD, rice-duck farming; OFC, open field cultivation; IPN, insect-proof net cultivation; AT, all treatments. ^∗∗^*P* < 0.01 and ^∗^*P* < 0.05.*

### Nitrogen Accumulation and Translocation

There were significant differences in the N accumulation and translocation between IN and OM. Compared to IN, WS increased the NT and the N accumulation at the MT by 8.72–12.64 and 5.39–7.62%, respectively, and BR increased those by 16.86–23.57 and 10.35–15.07%, respectively (**Table 7**). WS and BR had higher NTE and NCR than IN. However, the N accumulation, uptake ratio and rate were lower than those of IN from the TP to EG but higher than those of IN from the EG to MT.

**Table 7:** The effects of IPN, RD, and OM on the N accumulation and translocation characteristics of rice.

Treatments	N accumulation (kg.ha^-1^)					N uptake ratio (%)				N uptake rate (kg.ha^-1^.d^-1^)				N translocation from vegetative organs after heading		
	TP–ETC	ETC–EG	EG–HD	HD–MT	MT	TP–ETC	ETC–EG	EG–HD	HD–MT	TP–ETC	ETC–EG	EG–HD	HD–MT	NT (kg.ha^-1^)	NTE (%)	NCR (%)
OFC-CRIN	38.81b	34.64bc	68.41e	47.77e	189.63f	20.47a	18.27a	36.08d	25.19c	1.1089b	2.3093bc	2.5337e	0.9554e	47.50e	34.41e	47.91e
OFC-CRWS	35.11e	32.51d	81.22c	52.58d	201.42e	17.43b	16.14c	40.32b	26.11bc	1.0031e	2.1673d	3.0081c	1.0516d	52.23d	35.89d	48.29de
OFC-CRBR	36.28d	33.23d	88.82b	58.75c	217.08c	16.71bc	15.31d	40.91ab	27.07ab	1.0366d	2.2153d	3.2896b	1.1750c	58.24c	37.36bc	48.78cd
OFC-RDIN	40.01a	36.46a	77.10d	53.98d	207.55d	19.28a	17.57b	37.15c	26.00bc	1.1431a	2.4307a	2.8556d	1.0796d	55.06cd	36.58cd	49.11bc
OFC-RDWS	35.83de	33.44cd	92.14b	61.96b	223.37b	16.04c	14.97d	41.25a	27.74a	1.0237de	2.2293cd	3.4126b	1.2392b	62.02b	38.70ab	49.64b
OFC-RDBR	37.67c	35.07b	99.21a	66.87a	238.82a	15.78c	14.69d	41.54a	28.00a	1.0763c	2.3380b	3.6744a	1.3374a	68.04a	39.67a	50.31a
***F*-value**																
Mean	37.29	34.23	84.48	56.99	212.98	17.62	16.16	39.54	26.69	1.0653	2.2817	3.1290	1.1397	57.18	37.10	49.01
CP	68.21^∗^	35.34^∗^	227.88^∗∗^	269.70^∗∗^	298.19^∗∗^	55.86^∗^	232.66^∗∗^	22.52^∗^	37.28^∗^	68.21^∗^	35.34^∗^	227.88^∗∗^	269.70^∗∗^	182.70^∗∗^	69.00^∗^	168.64^∗∗^
FM	133.95^∗∗^	28.99^∗∗^	358.14^∗∗^	100.56^∗∗^	371.16^∗∗^	41.53^∗∗^	91.21^∗∗^	358.74^∗∗^	22.44^∗∗^	133.95^∗∗^	28.99^∗∗^	358.14^∗∗^	100.56^∗∗^	171.17^∗∗^	71.26^∗∗^	57.58^∗∗^
CP × FM	1.01	1.18	1.04	1.80	2.21	0.14	0.84	0.72	1.12	1.01	1.18	1.04	1.80	2.03	0.88	1.72
IPN-CRIN	37.76b	34.02b	65.77e	45.61e	183.16e	20.62a	18.58a	35.90b	24.90d	1.0789b	2.2680b	2.4359e	0.8771e	44.07e	32.88e	47.27d

IPN-CRWS	34.68e	31.95d	76.43cd	49.98d	193.04d	17.97b	16.55b	39.59a	25.89bc	0.9909e	2.1300d	2.8307cd	0.9612d	48.03d	34.02d	48.21c
IPN-CRBR	35.92cd	32.80cd	80.37bc	53.03c	202.12c	17.78b	16.23b	39.75a	26.24bc	1.0263cd	2.1867cd	2.9767bc	1.0198c	51.50c	34.90cd	48.58bc
IPN-RDIN	39.96a	35.85a	72.00de	50.42d	198.23cd	20.17a	18.10a	36.27b	25.45cd	1.1417a	2.3900a	2.6667de	0.9696d	50.91cd	35.34bc	48.51bc
IPN-RDWS	35.57de	33.00c	85.60ab	56.61b	210.78b	16.89bc	15.67bc	40.60a	26.86ab	1.0163de	2.2000c	3.1704ab	1.0887b	55.35b	36.29b	48.90b
IPN-RDBR	36.65c	33.36bc	92.35a	62.45a	224.81a	16.30c	14.84c	41.08a	27.77a	1.0471c	2.2240bc	3.4204a	1.2010a	61.49a	37.96a	49.52a
***F*-value**																
Mean	36.76	33.50	78.75	53.02	202.02	18.29	16.66	38.87	26.19	1.0502	2.2331	2.9168	1.0196	51.89	35.23	48.50
CP	57.20^∗^	31.01^∗^	31.46^∗^	347.22^∗∗^	110.28^∗∗^	41.54^∗^	23.16^∗^	3.38	51.30^∗^	57.20^∗^	31.01^∗^	31.46^∗^	347.22^∗∗^	340.22^∗∗^	142.34^∗∗^	275.09^∗∗^
FM	106.24^∗∗^	77.58^∗∗^	90.03^∗∗^	163.06^∗∗^	208.63^∗∗^	185.52^∗∗^	204.78^∗∗^	49.07^∗∗^	19.83^∗∗^	106.24^∗∗^	77.58^∗∗^	90.03^∗∗^	163.06^∗∗^	48.44^∗∗^	36.44^∗∗^	20.21^∗∗^
CP × FM	4.73^∗∗^	4.84^∗^	2.32	9.27^∗∗^	6.02^∗^	3.65	4.87^∗^	0.49	1.38	4.73^∗^	4.84^∗^	2.32	9.27^∗∗^	1.72	1.13	1.11

*TP, transplanting stage; ETC, effective tiller critical leaf stage; EG, elongation stage; HD, heading stage; MT, mature stage; NT, the amount of apparent N translocation from vegetative organs after the heading stage; NTE, apparent N translocation efficiency from vegetative organs after the heading stage; NCR, the contribution rate of the transferred N from vegetative organs to grain after the heading stage; IN, inorganic N fertilizer; WS, wheat straw return; BR, biogas residue return; CR, conventional rice farming; RD, rice-duck farming; OFC, open field cultivation; IPN, insect-proof net cultivation; CP, cultural practice (conventional rice farming and rice-duck farming); FM, fertilizer management (inorganic N fertilizer, wheat straw return and biogas residue return). Values within a column followed by different letters are significantly different at *P* < 0.05. ^∗∗^*P* < 0.01 and ^∗^*P* < 0.05.*

Significant differences were found in the N accumulation and translocation between CR and RD. Compared to CR, RD increased the NT and the N accumulation at the MT by 15.24–19.40 and 8.23–11.23%, respectively (**Table 7**). The NTE, NCR, N accumulation and uptake rate from the TP to MT of RD were higher than those of CR; the N uptake ratio of RD was lower than that of CR from the TP to EG but higher than that of CR from the EG to MT. Compared to OFC, the NT and the N accumulation at the MT of IPN were decreased by 7.22–11.57 and 3.41–6.89%, respectively. Meanwhile, IPN had a lower NTE, NCR, and N accumulation than OFC. Regarding the interaction between RD and OM, the NT, NTE, NCR, and N accumulation at the MT were higher than those of the interaction between CR and IN (**Table 7**). The interaction between RD and OM increased the NT and the N accumulation at the MT by 25.60–43.24 and 15.08–25.94%, respectively, which were higher than the single OM or RD.

Correlation analysis showed that the N accumulations from the TP to MT and during the MT were positively correlated with the rice yields of IN, WS, and BR, respectively (**Table 8**). The N accumulations from the EG to MT and during the MT were positively correlated with the rice yields under CR, RD, OFC and IPN, respectively. The positive correlations were found between the NT, the NTE, the N accumulation from the EG to MT, during the MT and the rice yield. These results suggested that the relatively strong N accumulation after the EG was important for achieving high yield.

**Table 8:** The effects of IPN, RD, and OM on the correlations between the yield and the N accumulation and translocation of rice.

Treatments	N accumulation					N translocation from vegetative organs after heading		
	TP–ETC	ETC–EG	EG–HD	HD–MT	MT	NT	NTE	NCR
IN (*n* = 12)	0.826^∗∗^	0.765^∗∗^	0.712^∗∗^	0.869^∗∗^	0.851^∗∗^	0.806^∗∗^	0.861^∗∗^	0.171
WS (*n* = 12)	0.741^∗∗^	0.674^∗^	0.872^∗∗^	0.839^∗∗^	0.881^∗∗^	0.865^∗∗^	0.935^∗∗^	-0.069
BR (*n* = 12)	0.803^∗∗^	0.757^∗∗^	0.804^∗∗^	0.792^∗∗^	0.817^∗∗^	0.855^∗∗^	0.894^∗∗^	0.346
CR (*n* = 18)	-0.229	-0.185	0.868^∗∗^	0.885^∗∗^	0.916^∗∗^	0.886^∗∗^	0.916^∗∗^	0.095
RD (*n* = 18)	-0.332	-0.218	0.834^∗∗^	0.897^∗∗^	0.903^∗∗^	0.926^∗∗^	0.954^∗∗^	0.161
OFC (*n* = 18)	-0.218	-0.034	0.906^∗∗^	0.911^∗∗^	0.918^∗∗^	0.882^∗∗^	0.898^∗∗^	0.240
IPN (*n* = 18)	-0.157	-0.143	0.848^∗∗^	0.880^∗∗^	0.889^∗∗^	0.904^∗∗^	0.926^∗∗^	0.187
AT (*n* = 36)	-0.094	0.057	0.875^∗∗^	0.898^∗∗^	0.911^∗∗^	0.907^∗∗^	0.930^∗∗^	0.240

*TP, transplanting stage; ETC, effective tiller critical leaf stage; EG, elongation stage; HD, heading stage; MT, mature stage; NT, the amount of apparent N translocation from vegetative organs after the heading stage; NTE, apparent N translocation efficiency from vegetative organs after the heading stage; NCR, the contribution rate of the transferred N from vegetative organs to grain after the heading stage; IN, inorganic N fertilizer; WS, wheat straw return; BR, biogas residue return; CR, conventional rice farming; RD, rice-duck farming; OFC, open field cultivation; IPN, insect-proof net cultivation; AT, all treatments. ^∗∗^*P* < 0.01 and ^∗^*P* < 0.05.*

### Nitrogen Utilization Efficiency

There were noticeable differences in NRE between IN and OM. Compared to IN, WS, and BR increased NRE by 12.06–14.88 and 23.11–31.01%, respectively (**Table 9**). WS and BR improved the NAE and NUG, while decreased the NPE and NGPE. Under OFC, the NDMPE of WS and BR was lower than that of IN (except for the treatment OFC-CRWS), while IPN showed an opposite pattern. These results indicated that the decomposition of organic matter could be affected by insect-proof net mulching.

**Table 9:** The effects of IPN, RD, and OM on the rice N utilization efficiency.

Treatments	NRE (%)	NAE (kg.kg^-1^)	NPE (kg.kg^-1^)	NGPE (kg.kg^-1^)	NDMPE (kg.kg^-1^)	NUG (100 kg.kg^-1^)
OFC-CRIN	29.51f	11.39d	38.60a	49.34a	86.22b	2.03e
OFC-CRWS	33.44e	12.25c	36.64ab	47.74ab	86.99ab	2.10de
OFC-CRBR	38.66c	13.27b	34.33bc	45.71cd	84.48c	2.19bc
OFC-RDIN	35.48d	12.31c	34.70bc	46.42bc	88.57a	2.15cd
OFC-RDWS	40.76b	13.39b	32.85cd	44.57d	88.42a	2.24b
OFC-RDBR	45.91a	14.10a	30.73d	42.59e	88.26a	2.35a
***F*-value**						
Mean	37.29	12.79	34.64	46.06	87.16	2.18
CP	298.19^∗∗^	81.60^∗^	40.75^∗^	58.13^∗^	208.50^∗∗^	62.76^∗^
FM	371.16^∗∗^	86.38^∗∗^	39.23^∗∗^	90.66^∗∗^	2.85	96.55^∗∗^
CP × FM	2.21	0.64	0.05	0.11	2.04	0.84

IPN-CRIN	27.35e	10.61c	38.77a	49.81a	83.03c	2.01c
IPN-CRWS	30.65d	11.25bc	36.70ab	48.26ab	84.70bc	2.07bc
IPN-CRBR	33.67c	12.06ab	35.82abc	47.29ab	86.39ab	2.11bc
IPN-RDIN	32.38cd	11.49bc	35.58abc	47.39ab	87.37a	2.11bc
IPN-RDWS	36.56b	12.22ab	33.46bc	45.60bc	87.99a	2.19ab
IPN-RDBR	41.24a	12.98a	31.49c	43.75c	87.46a	2.29a
***F*-value**						
Mean	33.64	11.77	35.30	47.02	86.16	2.13
CP	110.28^∗∗^	22.15^∗^	10.97	20.98^∗^	27.64^∗^	19.08^∗^
FM	208.63^∗∗^	25.61^∗∗^	15.41^∗∗^	41.94^∗∗^	6.98^∗^	45.19^∗∗^
CP × FM	6.02^∗^	0.02	0.51	1.57	6.04^∗^	2.87

*The total N uptake of plants in the area without N application was 101.1 kg⋅ha**^-^**^1^; the rice yield in the area without N application was 5.94 t⋅ha**^-^**^1^. NRE, N recovery efficiency; NAE, N agronomic efficiency; NPE, N physiological efficiency; NGPE, N grain production efficiency; NDMPE, N dry matter production efficiency; NUG, N uptake per 100 kg of grains; IN, inorganic N fertilizer; WS, wheat straw return; BR, biogas residue return; CR, conventional rice farming; RD, rice-duck farming; OFC, open field cultivation; IPN, insect-proof net cultivation; CP, cultural practice (conventional rice farming and rice-duck farming); FT, fertilizer management (inorganic N fertilizer, wheat straw return and biogas residue return). Values within a column followed by different letters are significantly different at *P* < 0.05. ^∗∗^*P* < 0.01 and ^∗^*P* < 0.05.*

Compared to CR, RD significantly increased the NRE by 18.39–22.48% (**Table 9**). RD also increased the NAE, NDMPE, and NUG but decreased the NPE and NGPE. However, the NRE of IPN was 7.32–12.91% lower than that of OFC. Compared to OFC, IPN decreased the NAE and NUG but increased the NPE and NGPE. The NDMPE of IPN was lower than that of OFC (except for the treatment IPN-CRBR). The interaction between RD and OM increased the NRE by 33.67–55.57% (**Table 9**). Meanwhile, the interaction between RD and OM had higher NAE, NDMPE, and NUG but lower NPE and NGPE compared to the interaction between CR and IN.

Correlation analysis indicated that there were positive correlations between the NRE, the NAE and the rice yield under WS and BR (**Table 10**). The rice yields under IN, CR, RD, OFC, and IPN were positively correlated with the NRE, NAE, and NDMPE but were negatively correlated with the NPE and NGPE. The rice yield had positive correlations with NRE, NAE, NDMPE, and NUG but had negative correlations with the NPE and NGPE, which suggested that increasing the NRE and NAE were beneficial to improve the rice yield.

**Table 10:** The effects of IPN, RD, and OM on the correlations between the rice yield and the N utilization efficiency.

Treatments	NRE	NAE	NPE	NGPE	NDMPE	NUG
IN (*n* = 12)	0.800^∗∗^	0.716^∗∗^	-0.703^∗^	-0.614^∗^	0.614^∗^	0.675^∗^
WS (*n* = 12)	0.847^∗∗^	0.829^∗∗^	-0.493	-0.649^∗^	0.655^∗^	0.372
BR (*n* = 12)	0.772^∗∗^	0.807^∗∗^	-0.603^∗^	-0.560	0.557	0.423
CR (*n* = 18)	0.874^∗∗^	0.850^∗∗^	-0.647^∗∗^	-0.682^∗∗^	0.682^∗∗^	0.284
RD (*n* = 18)	0.877^∗∗^	0.845^∗∗^	-0.694^∗∗^	-0.720^∗∗^	0.722^∗∗^	0.065
OFC (*n* = 18)	0.887^∗∗^	0.856^∗∗^	-0.829^∗∗^	-0.794^∗∗^	0.794^∗∗^	0.156
IPN (*n* = 18)	0.861^∗∗^	0.821^∗∗^	-0.734^∗∗^	-0.739^∗∗^	0.732^∗∗^	0.514^∗^
AT (*n* = 36)	0.887^∗∗^	0.878^∗∗^	-0.740^∗∗^	-0.761^∗∗^	0.760^∗∗^	0.409^∗^

*NRE, N recovery efficiency; NAE, N agronomic efficiency; NPE, N physiological efficiency; NGPE, N grain production efficiency; NDMPE, N dry matter production efficiency; NUG, N uptake per 100 kg of grains; IN, inorganic N fertilizer; WS, wheat straw return; BR, biogas residue return; CR, conventional rice farming; RD, rice-duck farming; OFC, open field cultivation; IPN, insect-proof net cultivation; AT, all treatments. ^∗∗^*P* < 0.01 and ^∗^*P* < 0.05.*

## Discussion

### Dry Matter Accumulation and Translocation Characteristics, and Their Relationships with Rice Yield

Dry matter accumulation and translocation could limit rice yield, as shown by the dry matter accumulation and dry matter translocation ratio to grains (San-oh et al., 2004). In the present study, WS and BR decreased the dry matter accumulation from the TP to EG but increased the dry matter accumulation from the EG to MT (**Table 5**). This was primarily because the microorganisms increased rapidly and consumed a portion of the mineral N after returning wheat straw into the soil (Chen et al., 2014). On the other hand, wheat straw decomposition produced reducing harmful substances at an earlier stage, which influenced rice root growth (Bradford and Peterson, 2000). However, at later stage, the degradation of soil microorganisms produced large amounts of organic matter and physiological activators, which improved soil fertility (Hao et al., 2010). OM didn’t benefit rice population development at an earlier stage but was beneficial for the development at middle and later stages. The results were similar to the findings of Ye et al. (2008) that the application of wheat straw could lengthen photosynthetic time, improve photosynthetic efficiency and promote the translocation of photosynthetic products to grains. However, the researches by Rao and Mikkelsen (1976) showed that straw return had adverse effects on rice growth and nutrition, which might be due to the different planting methods.

Rice-duck farming improved the leaf area index and effective leaf area ratio in the middle and lower parts of rice and enhanced the leaf photosynthetic ability, which provided a foundation for high yield (Liu et al., 2015). In this study, RD promoted the dry matter accumulation and translocation, and increased the rice yield (**Table 5**; **Figure 2**), which might be due to the fact that the feeding habits and activities of the ducks stimulated rice growth; on the other hand, the intertillage and manure fertilizer promoted the formation of a strong source and efficient flow and resulted in great sink activity. However, these results were different from the reports of Zhang et al. (2010) who showed that the organic rice yield of RD was lower than that of CR and was not beneficial to improve the rice yield, and the differences were mainly due to the different planting density. Facing a large area of crop lodging due to excessive fertilization in rice production, RD provide a new farming mode in which ducks play a role in controlling weeds, fertilizing rice plants, enhancing lodging resistance of the rice stalks and easing yield loss (Wang et al., 2008).

The major effect of shading is the reduction of light intensity (Chan and Mackenzie, 1972). Shading resulted in a decrease in rice yield (Moula, 2009). In the study, IPN increased the leaf SPAD values of rice in a netting house (**Figure 3**), while decreased the *Pn*, dry matter accumulation and translocation, and rice yield (**Tables 4** and **5**; **Figure 2**), possibly because IPN reduced the wind speed, air motion, CO_2_ content and light intensity (**Figure 1**; **Table 2**), which inhibited photosynthesis and dry matter accumulation, and was not conducive to the rice production. These results were similar to the reports of Wang L. et al. (2015) who showed that shading increased the flag leaf chorophyll content but decreased the net photosynthetic rate and grain yield. The interaction between RD and OM promoted the dry matter accumulation from the EG to MT, the dry matter translocation and rice yield (**Table 5**; **Figure 2**), which might lie in the activities of the ducks in the field, which potentially accelerated the decomposition of wheat straw and biogas residue and promoted the release of nutrients.

The relatively strong light absorption, translocation, and utilization ability of flag leaves promoted dry matter accumulation, and the relatively high dry matter accumulation at a late stage was the basis for grain filling (Zhang et al., 2003). In the study, the dry matter accumulation and translocation were positively correlated with the rice yield (**Table 6**). The results were in conformity with the findings of Deng et al. (2015) that the rice yield was positively correlated with the dry matter accumulation after panicle initiation stage and the post-anthesis transfer of accumulated dry matter into grain. Therefore, an increase in dry matter accumulation was helpful for the improvement of rice production.

### Nitrogen Accumulation and Translocation, Nitrogen Utilization Efficiency, and their Relationships with Rice Yield

N is an indispensable nutrient for rice growth, and the supply of N strongly regulates rice yield (Yousaf et al., 2016), N absorption and translocation. The chlorophyll content of rice leaves is an active component of N utilization and is closely related to leaf photosynthetic ability (Shiratsuchi et al., 2006; Ata-Ul-Karim et al., 2016). In this study, WS and BR decreased the leaf SPAD values, *Pn*, N accumulation before the EG and N translocation but increased the N accumulation from the EG to MT and N translocation (**Figure 3**; **Tables 4 and 7**). The results might be that the high C/N ratio of wheat straw promoted the mass propagation of microorganisms, which competed with rice for N after being returned to the field. This led to a decrease in the amount of soil N taken up by the plants at earlier stage (Xiong et al., 2015). Afterward, organic matter gradually decomposed and released the nutrient substances, which was beneficial for the N absorption of rice. OM promoted the cycling of organic matter and relieved environmental problems resulting from the use of large quantities of chemical N fertilizer.

Rice-duck farming played an important regulatory role in alleviating nutrient shortages. In this study, RD increased the amount of N accumulation and translocation (**Table 7**), which might be that the return of duck manure to the soil and the activities of the ducks increased the amount of N taken up by the plants. The results were similar to the findings of Yu et al. (2009) and Zhang (2012). In this study, because of the insect-proof net mulching, IPN decreased the N accumulation and translocation. However, the interaction between RD and OM increased the N accumulation from the EG to MT and the N translocation, which were higher than single RD or OM (**Table 7**), and this might be due to the dual influences of RD and OM. Sun et al. (2012) found that the rice yield was significantly and positively correlated with N accumulation and translocation. In this study, the N accumulation from the EG to MT and the N translocation were positively correlated with the rice yield (**Table 8**). The results showed that the N accumulation from the EG to MT played a vital role in N accumulation and the greater N accumulation between the EG and the MT corresponded with the higher rice yield.

The N utilization efficiency of rice involves carbohydrate metabolism, nutrient signal transmission, and protein synthesis and degradation within plants as well as regulatory feedback via bioactivators (Chen et al., 2003). Therefore, it is important to study the N utilization efficiency of plants with respect to rice growth and yield (Massel et al., 2016). In the study, OM increased NRE, NAE and NUG but decreased NPE and NGPE (**Table 9**), which was similar to the reports of Yan et al. (2015) that rice N accumulation, NRE and NAE were significantly increased under WS. In addition, the present study showed that IPN decreased NRE and NAE and was associated with a risk of reducing nutrient utilization. However, RD and the interaction between RD and OM had the higher NRE, NAE, and NUG (**Table 9**). The results indicated that OM, RD and the interaction between RD and OM provided the methods for accumulating the high N content.

In this study, NRE, NAE, NDMPE, and NUG were positively correlated with rice yield but negatively correlated with NPE and NGPE (**Table 10**), which were consistent with the reports of Ntanos and Koutroubas (2002) and Li et al. (2014). However, Peng et al. (2006) noted that the internal N utilization efficiency (NGPE) was supplementary to the NAE. The results were different from the present study, the differences might be due to the fact that the greater increment in the rice N accumulation than in the rice yield observed in the present study. Higher NRE, NAE, NDMPE, and NUG and lower NPE and NGPE corresponded with higher rice yield. Accordingly, it is possible to increase dry matter and N accumulation while achieving high yield and high N utilization efficiency.

## Conclusion

Organic matter return increased the dry matter accumulation, N absorption and utilization after EG and also improved the NRE and rice yield due to the high photosynthesis. The magnitude of the increase in rice yield was greater for BR than for WS. RD had the greater rice leaf photosynthesis, dry matter and N accumulation, dry matter and N translocation, and NRE, which finally resulted in the higher rice yield. However, insect-proof nets decreased the intensity of the radiation reaching the plants and therefore were not beneficial for the dry matter accumulation and translocation, N accumulation and utilization, NRE and rice yield. The interaction between RD and OM promoted the leaf *Pn*, dry matter accumulation, N absorption, NRE and rice yield of rice, and the effect of the interaction between RD and OM was better than that of single RD or OM. In addition, the dry matter accumulation, the N accumulation from the EG to MT, dry matter and N translocation were positively correlated with the rice yield. Therefore, OM, RD and the interaction between RD and OM contribute to increasing the rice yield, which can relieve the pressure of global food.

## Author Contributions

QW and XL conceived and designed the research. XL, GX, and YH carried out the experiments. QW, XL, and GX analyzed experimental data. XL and QW wrote the main manuscript text. All authors reviewed the manuscript.

## Conflict of Interest Statement

The authors declare that the research was conducted in the absence of any commercial or financial relationships that could be construed as a potential conflict of interest.

## Abbreviations

BR: biogas residue return
*Ci*: intercellular CO_2_ concentration
CR: conventional rice farming
DCR or NCR: the contribution rate of the transferred dry matter or N respectively from vegetative organs to grain after the heading stage
DT or NT: the amount of apparent dry matter or N translocation, respectively, from vegetative organs after the heading stage
DTE or NTE: apparent dry matter or N translocation efficiency, respectively, from vegetative organs after the heading stage
30 DAH: 30 days after heading
EG: elongation stage
ETC: effective tiller critical leaf stage
*Gs*: stomatal conductance
HD: heading stage
IN: inorganic N fertilizer
IPN: insect-proof net cultivation
MT: mature stage
NAE: N agronomic efficiency
NDMPE: N dry matter production efficiency
NGPE: N grain production efficiency
NPE: N physiological efficiency
NRE: N recovery efficiency
NUG: N uptake per 100 kg of grains
OFC: open field cultivation
OM: organic matter return
*Pn*: net photosynthetic rate
RD: rice-duck farming
TP: transplanting stage
*Tr*: transpiration rate
WS: wheat straw return
